# Re-refinement of 4g4a: room-temperature X-ray diffraction study of cisplatin and its binding to His15 of HEWL after 14 months chemical exposure in the presence of DMSO

**DOI:** 10.1107/S2053230X16000856

**Published:** 2016-02-19

**Authors:** Simon W. M. Tanley, Antoine M. M. Schreurs, Loes M. J. Kroon-Batenburg, John R. Helliwell

**Affiliations:** a School of Chemistry, Faculty of Engineering and Physical Sciences, University of Manchester, Brunswick Street, Manchester M13 9PL, England; b Crystal and Structural Chemistry, Bijvoet Center for Biomolecular Research, Faculty of Science, Utrecht University, Padualaan 8, 3584 CH Utrecht, The Netherlands

**Keywords:** improved diffraction resolution, raw diffraction images., re-refinement, cisplatin, histidine, addendum

## Abstract

A re-refinement of 4g4a, the room-temperature X-ray diffraction study of cisplatin and its binding to His15 of HEWL after 14 months chemical exposure in the presence of DMSO is published as an addendum to Tanley *et al.* [(2012), *Acta Cryst.* F**68**, 1300–1306]. This example illustrates the benefits of sharing raw diffraction images, as well as structure factors and molecular coordinates, as the diffraction resolution of the study is now much improved at 1.70 Å.

## Abstract 2

An addendum is published to Tanley *et al.* [(2012), *Acta Cryst.* F**68**, 1300–1306].

We have re-refined our previously published room temperature crystal structure of cisplatin binding to hen egg lysozyme (PDB entry 4g4a; Tanley *et al.*, 2012 ▸) at a resolution of 1.7 Å. This structure was originally refined by us at 2.4 Å resolution and subsequently re-refined by Shabalin *et al.* (2015 ▸) to 2.0 Å resolution. Both studies extended the diffraction resolution by reprocessing subsets of the same data set’s raw diffraction images [originally held at Utrecht University; Tanley *et al.* (2013 ▸) and which are now accessible at the University of Manchester (Tanley & Helliwell, 2015 ▸)]. Shabalin *et al.* (2015 ▸) (PDB code 4yen) interpreted the platinum coordination spheres as [PtCl_3_His15] for the platinum ion (Pt^δ^) coordinated to N^δ^ of His15 and [PtCl_2_His15Arg14] for the second platinum ion (Pt^∊^) bound to His15-N^∊^. A re-refinement of 4g4a at the extended resolution of 1.7 Å presented here gives improved clarity, and a small spread of *B* factors, of the Pt ligand assignments which are [PtClNH_3_NH_3_His15] for Pt^δ^, and [PtClNH_3_His15Arg14] for Pt^∊^; see Fig. 1 ▸. Details are provided in the Supporting Information.

The following references are cited in the Supporting Information for this article: Afonine *et al.* (2012 ▸); Casini *et al.* (2007 ▸); Diederichs & Karplus (2013 ▸); Joosten *et al.* (2014 ▸); Murshudov *et al.* (1997 ▸); Schreurs *et al.* (2010 ▸); Tanley, Schreurs, Kroon-Batenburg, Meredith *et al.* (2012 ▸); Tanley *et al.* (2015 ▸). 

## Supplementary Material

PDB reference: re-refinement of 4g4a, 5hll

Supporting Information.. DOI: 10.1107/S2053230X16000856/no5101sup1.pdf

## Figures and Tables

**Figure 1:**
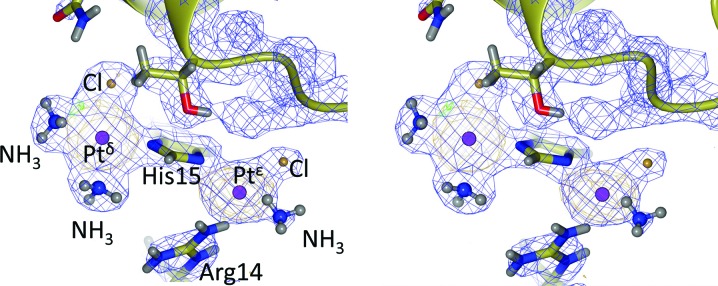
Stereo image of the cisplatin coordination to His15. A platinum ion is bound to both the N^δ^ and N^∊^ sides of the imidazole ring with ligands (two ammines and one Cl atom at Pt^δ^, and one chlorine, one ammine and the Arg14 side chain at Pt^∊^). Electron-density maps: 2*F*_o_ − *F*_c_ (in blue at 1.0 r.m.s.), *F*_o_ − *F*_c_ (in green at 5.0σ) and anomalous difference Fourier map (in orange at 3.0σ) of the final model. The two platinum ions are depicted in purple, the Cl atoms in brown, the N atoms in blue, O atoms in red and H atoms in charcoal grey. The orientation of each ammine’s three H atoms and of the Arg14 side-chain H atoms at 1.7 Å diffraction resolution are obviously approximate (the Arg14 side-chain nitrogen closest to the Pt^∊^ in particular would present a lone pair of electrons to the metal atom). This figure was prepared using *CCP4mg* (McNicholas *et al.*, 2011 ▸).

## References

[bb1] Afonine, P. V., Grosse-Kunstleve, R. W., Echols, N., Headd, J. J., Moriarty, N. W., Mustyakimov, M., Terwilliger, T. C., Urzhumtsev, A., Zwart, P. H. & Adams, P. D. (2012). *Acta Cryst.* D**68**, 352–367. 10.1107/S0907444912001308 PMC3322595 22505256

[bb2] Casini, A., Mastrobuoni, G., Temperini, C., Gabbiani, C., Francese, S., Moneti, G., Supuran, C. T., Scozzafava, A. & Messori, L. (2007). *Chem. Commun.* 156–158. 10.1039/b611122j 17180231

[bb3] Diederichs, K. & Karplus, P. A. (2013). *Acta Cryst.* D**69**, 1215–1222. 10.1107/S0907444913001121 PMC3689524 23793147

[bb4] Joosten, R. P., Long, F., Murshudov, G. N. & Perrakis, A. (2014). *IUCrJ*, **1**, 213–220. 10.1107/S2052252514009324 PMC4107921 25075342

[bb5] McNicholas, S., Potterton, E., Wilson, K. S. & Noble, M. E. M. (2011). *Acta Cryst.* D**67**, 386–394. 10.1107/S0907444911007281 PMC3069754 21460457

[bb6] Murshudov, G. N., Vagin, A. A. & Dodson, E. J. (1997). *Acta Cryst.* D**53**, 240–255. 10.1107/S0907444996012255 15299926

[bb7] Schreurs, A. M. M., Xian, X. & Kroon-Batenburg, L. M. J. (2010). *J. Appl. Cryst.* **43**, 70–82.

[bb8] Shabalin, I., Dauter, Z., Jaskolski, M., Minor, W. & Wlodawer, A. (2015). *Acta Cryst.* D**71**, 1965–1979. 10.1107/S139900471500629X PMC4556316 26327386

[bb9] Tanley, S. W. M. , Diederichs, K., Kroon- Batenburg, L. M. J. , Levy, C., Schreurs, A. M. M. & Helliwell, J. R. (2015). *Acta Cryst.* D**71**, 1982–1983. 10.1107/S1399004715014340 26327388

[bb10] Tanley, S. W. M. & Helliwell, J. R. (2015). https//www.escholar.manchester.ac.uk/uk-ac-man-scw215887. 10.15127/1.215887.

[bb11] Tanley, S. W. M., Schreurs, A. M. M., Helliwell, J. R. & Kroon-Batenburg, L. M. J. (2013). *J. Appl. Cryst.* **46**, 108–119. 10.1107/S0021889812044172 PMC3547227 23396873

[bb12] Tanley, S. W. M., Schreurs, A. M. M., Kroon-Batenburg, L. M. J. & Helliwell, J. R. (2012). *Acta Cryst.* F**68**, 1300–1306. 10.1107/S1744309112042005 PMC3515368 23143236

[bb13] Tanley, S. W. M., Schreurs, A. M. M., Kroon-Batenburg, L. M. J., Meredith, J., Prendergast, R., Walsh, D., Bryant, P., Levy, C. & Helliwell, J. R. (2012). *Acta Cryst.* D**68**, 601–612. 10.1107/S0907444912006907 22525758

