# Supplementary material for: Re-refinement of 4g4a: room-temperature X-ray diffraction study of cisplatin and its binding to His15 of HEWL after 14 months chemical exposure in the presence of DMSO
Source: Acta Crystallogr F Struct Biol Commun. 2016 Feb 19;72(Pt 3):253–4. doi: 10.1107/S2053230X16000856 (PMC4774887; doi:10.1107/S2053230X16000856)
Supplement: Supplementary file 1 [file f-72-00253-sup1.pdf]

STRUCTURAL BIOLOGY
COMMUNICATIONS

Volume 72 (2016)

Supporting information for article:

Re-refinement of 4g4a: room-temperature X-ray diffraction study of cisplatin and its binding to His15 of HEWL after 14 months chemical exposure in the presence of DMSO

Simon W. M. Tanley, Antoine M. M. Schreurs, Loes M. J. Kroon-Batenburg and John Helliwell

Following our response (Tanley et al 2015) to the article of Shabalin et al. (2015), which reported a re-refinement of our structure of hen egg lysozyme in complex with cis-platin (pdb entry 4g4a, Tanley et al 2012a)), we have corrected the solute molecules that were highlighted. We have also reprocessed our raw diffraction data images, which we had made publicly available (Tanley et al 2013), and extended the resolution from 2.4Å to 1.7 Å (Table 1) using EVAL (Schreurs et al 2010). The omit electron density maps confirm the binding of platinum to both the His15 ND and NE sides of the imidazole ring as also modelled by Shabalin et al (2015) at 2.0Å (PDB code 4yen) and our original paper Tanley et al 2012a and its associated PDB file 4g4a.

At this improved diffraction resolution of 1.7Å for the His15 ND side of the imidazole ring the omit electron density map showed three peaks at the expected geometric positions for the platinum ligands forming a square planar coordination arrangement. The omit map peak heights were 12.2σ, 9.8σ and 8.0σ and from which the assignment of chloride and two ammines respectively were made. The His15 NE position cisplatin binding site omit map showed one clear ligand and, taking account of its peak height (adjusted for the Pt NE occupancy) was assigned to a chloride; another of this platinum atom's ligands is the nitrogen of the Arg14 side chain and, in a later step, involving an omit map, we assigned an ammine. PHENIX_REFINE (Afonine et al 2012) was used for model refinement. Table 2 gives the cisplatin ligand details to the His15 ND and NE sides. The final refined model statistics are given in Table 1. We used the paired refinement method of assessment of the diffraction resolution of Diederichs and Karplus (2013) both manually and also as implemented automatically in Joosten et al (2014). These calculations indicated an improvement of the 1.7Å model compared to the 2.4 Å model. The 1.7Å model showed a better agreement against the 2.0Å diffraction data with initial and final Rwork and Rfree values respectively 17.5% vs 18.2% and 20.2% vs 20.4%. We also note that by adding data between 2.0 and 1.7 Å resolution, the number of reflections increased by 26150. Of these 7817 had an $I/\sigma > 1$, and 2317 had an $I/\sigma > 2$. So, significant new information has been included by adding these data beyond 2.0Å.

For this 1.7Å crystal structure we assessed the stability of the B factor value estimates in Table 2 of our final re-refined structure by refining it in Refmac (Murshudov et al 1997) and the results are displayed in Table 3a. The B value estimates are similar in the two refinement programs. Likewise to assess the stability of the 4yen assigned ligands' B factors we refined

4yen against our new 1.7Å dataset. The results are shown in Table 3b and shows that three of the chloride assignments of the cisplatin, two on the ND side and one on the NE side, have large B factors at both resolutions (the original 2.0Å and now also at 1.7Å resolution) indicative of a ligand with a smaller number of electrons being present, and to which we have instead assigned an ammine at each of those three 4yen chloride positions.

In Table 4, for clarity about what has finally changed in the coordination arrangement of the two platinum atoms of this new study versus the two previous studies, we compare the cisplatin ligand assignments in our 1.7Å crystal structure with 4yen (at 2.0Å) and with 4g4a (at 2.4Å) along with their occupancy values and B factor estimates. The cisplatin ligand occupancies used in this 1.7Å crystal structure are the same as in 4yen. The spread of B factor values in this 1.7Å crystal structure for the cisplatin ligands is now quite small, indicating that reasonable ligand assignments have now been made. We would note that the challenge of differentiating between ammine and chloride, respectively ten versus 17 electrons, is easier at the higher resolution.

Overall, this new model for the platinum coordination on both sides of the His15 imidazole ring at 1.7 Å showed two ammine ligands and one chlorine at the ND side and one chlorine, one ammine and the Arg 14 side chain at the NE side. These ligands refined well i.e. with a relatively smaller spread of B factors for each of the ligands when restrained to the same occupancy value as their respective platinum atoms, compared with the three chlorine atom ligand assignments in 4yen.

Our histidine binding studies of these platin compounds under a wide range of chemical conditions, including high salt and salt free, have revealed a tendency of cisplatin to transform and so a partial i.e. split occupancy of chlorine and ammine at each ligand position to the platinum cannot be ruled out at this diffraction resolution of 1.7Å.

Table S1 Data processing and final model refinement details

PDB id	5HLL(was 4g4a)
Data collection temperature (K)	300
**Data reduction**	
Space group	$P4_32_12$
Unit cell parameters (Å)	a=b= 79.14<br>c= 38.0
Detector to crystal distance (mm)	39.19
Observed reflections	202726
Unique reflections	13804
Resolution (Å) (last shell)	35.39 – 1.699 (1.728 –1 .699)
Completeness (%)	99.68 (93.86)
Rmeas (%)	0.132 (2.428)
<I/sigma(I)>	9.8 (0.56)
Multiplicity	6.7 (4.5)
Wilson B-factor (Å^2)<br>19.0	
Number of protein atoms (exc H)	1031
Average B factor (Å^2) for protein atoms	22
Number of ligands or ions (ie 1 DMS; 2 Cls; 1 Na); exc cisplatin (see Table 2).	4

Average B factor (Å^2) for ligand atoms 33	
Number of water molecules 32	
Average B factor (Å^2) for water molecules	24
R factor/ R free (%)	18.0/22.4
RMSD bonds (Å)/ Angles (°)	0.013/1.3
**Ramachandran values (%)**	
Most favoured	98.47
Additional allowed	1.53
Disallowed	0.0

Table S2 The 1.7Å final model refined occupancies and B factor values at the cisplatin binding sites with chemical assignments made on the basis of the omit electron density map.

Atom	Occupancy	B factor $^{\$}$	Distance to the platinum ion (Å)
Pt^{δ}	0.8*	33	
Cl	0.8*	27	2.4
NH_3 (end position)	0.8*	27^{+}	2.1
NH_3 (trans to the Cl)	0.8*	16^{+}	2.1
His15ND	1.0	27	2.1
Pt^{ε}	0.4*	32	
Cl	0.4*	23	2.3
NH_3 (end position)	0.4*	17^{+}	2.1
Arg14N	0.4*	48	2.3
His15 NE	1.0	21	2.4

*The standard uncertainties on the occupancies are probably about 10% ie 0.1 (for an evaluation of these standard uncertainties on the platinum atoms in this compound's binding to His15 for a very similar range of diffraction resolution datasets see Tanley et al 2012b).

$These values' standard uncertainties are probably ~ +/- 5 $Å^2$ (Tanley et al 2012b).

+ The B factor quoted is for the nitrogen atom; the attached hydrogens are very similar but slightly larger (by ~ 5 $Å^2$).

Table S3 Comparion of B factors

(a) Comparison of the cisplatin ligand atom B factors refined at 1.7Å using Phenix Refine (as in Table 2) with the same PDB file from Phenix Refine taken into Refmac. The atom occupancies (Table 2) are the same between the two.

Atom	Phenix Refine B factors	Refmac B factors
Pt^{δ}	33	32
Cl	27	29
NH_3 (end position) nitrogen atom	27	25

NH_3 (trans to the Cl) nitrogen atom	16	15
His15ND	27	27
Pt^{ε}	32	31
Cl	23	24
NH_3 (end position) nitrogen atom	17	16
Arg14N	48	43
His15 NE	21	24

Comparison of the 4yen cisplatin ligand atom B factors (Shabalin et al 2015) with the 4yen now refined (Refmac) against the 1.7Å data set presented with this paper. The atom occupancies are the same between the two.

Atom	4yen's cisplatin Pt and ligand assignment, occupancy and B factor	4yen cisplatin Pt and ligands refined against the 1.7Å new data set presented with this paper.
Pt^{δ}	41	32
Cl	36	29
Cl	52	48
Cl	45	38
His15ND	32	28
Pt^{ε}	38	31
Cl	29	24
Cl	38	36
Arg14N	41	38
His15NE	32	28

Table S4 Comparison of the final 1.7Å cisplatin ligand atom B factors and ligand atom assignments with 4yen and 4g4a.

Cisplatin site	The 1.7 Å crystal structure (Phenix Refine occupancy and B factors ie as Table 2)	4yen's cisplatin ligand assignment, occupancy and B factor	4g4a's cisplatin ligand assignment, occupancy and B factor*
Pt^{δ}	0.8, 33	0.8,41	0.8,37
	Cl ,0.8, 27	Cl, 0.8,36	N, 1.0, 28
	NH_3 (end position) nitrogen atom 0.8, 27	Cl, 0.8,52	N, 1.0, 30
	NH_3 (trans to the Cl) nitrogen atom 0.8, 16	Cl, 0.8,45	Cl, 1.0, 49
His15ND	1.0, 27	1.0, 32	1.0, 33
Pt^{ε}	0.4, 32	0.4,38	0.5,47
	Cl , 0.4, 23	Cl, 0.4,29	N, 1.0, 40
	NH_3 (end position) nitrogen atom , 0.4, 17	Cl, 0.4,38	N, 1.0, 46
Arg14N	1.0, 48	1.0,41	1.0, 36
His15 NE	1.0, 21	1.0, 32	1.0,31

* In 4g4a the ligand atom occupancies were not set equal to the Pt atom occupancy to which they were linked. Also the ammines were each approximated by a nitrogen atom alone. The 4g4a is included in this table to explicitly show that these two aspects have now been rectified.

Acknowledgements

We thank Shabalin et al (2015) for their valuable critique to which we promptly responded (Tanley et al 2015). We have modified their suggestion to use diffraction data to 2.0 Å and have reprocessed our raw diffraction images to 1.7Å resolution and used more of the diffraction images than they did; our modification of their suggestion does not alter the fact that their observation on this aspect of our study was a good one and we thank them for it. We again recognise that we have built upon the seminal crystallographic with mass spectrometry data

study of Casini et al 2007, and who we also thank. We are very grateful to Dr Kay Diederichs for detailed discussions.

References

Afonine, P.V. , Grosse-Kunstleve, R.W. , Echols, N., Headd, J.J. , Moriarty, N.W. , Mustyakimov, M. , Terwilliger, T.C. , Urzhumtsev, A. , Zwart, P.H. and Adams. P.D. (2012) *Acta Cryst* D68, 352-67 .

Casini, A., Mastrobuoni, G., Temperini, C., Gabbiani, C., Francese, S.,
Moneti, G., Supuran, C. T., Scozzafava, A. & Messori, L. (2007).
Chem. Commun., 156–158.

Diederichs, K. and Karplus, P. A. (2013) Acta Cryst D**69**, 1215–1222.

Joosten, R. P. , Long, F., Murshudov G. N. , and Perrakis, A. (2014) *IUCrJ* 1, 213-20.

McNicholas, S. , Potterton, E. , Wilson, K. S. and Noble, M. E. M. (2011) Acta Cryst. D67, 386-394.

Murshudov, G.N., Vagin, A.A. and Dodson, E.J. (1997) Acta Cryst. **D53**, 240-255.

Schreurs, A. M. M., Xian, X. & Kroon-Batenburg, L. M. J. (2010). J.
Appl. Cryst. 43, 70–82.

Shabalin, I., Dauter, Z., Jaskolski, M., Minor, W. & Wlodawer, A. (2015) *Acta Cryst.* D71, 1965–1979.

Tanley, S. W. M. , Schreurs, A. M. M. , Kroon-Batenburg, L. M. J. and Helliwell, J. R. (2012a) *Acta Cryst.*. F68, 1300–1306.

Tanley, S.W.M., Schreurs, A.M.M., Kroon-Batenburg, L.M.J., Meredith, J., Prendergast, R., Walsh, D., Bryant, P., Levy, C. and Helliwell, J.R. (2012b) *Acta Cryst.* D68, 601-612.

Tanley, S. W. M. , Schreurs, A. M. M., Helliwell, J. R. and Kroon-Batenburg, L. M. J. *J. Appl. Cryst.* (2013). 46, 108-119.

Tanley, S. W. M. , Diederichs, K., Kroon- Batenburg, L. M. J. , Levy, C., Schreurs. A. M. M. and Helliwell, J. R. (2015). *Acta Cryst.* D71, 1982–1983.
